# Flow-Line-Reducing Tetrahedral Metal Effect Pigments for Injection Molding: A Yield-Rate-Improved Particle Manufacturing Method Based on Soft UVImprint Lithography

**DOI:** 10.3390/polym17192708

**Published:** 2025-10-08

**Authors:** Nils Maximilian Demski, Holger Seidlitz, Felix Kuke, Oliver Niklas Dorn, Janina Zoglauer, Tobias Hückstaedt, Paul Hans Kamm, Francisco García-Moreno, Noah Kremp, Christian Dreyer, Dirk Oberschmidt

**Affiliations:** 1 Research Division Polymeric Materials and Composites PYCO, Fraunhofer Institute for Applied Polymer Research IAP, 15745 Wildau, Germany; holger.seidlitz@iap.fraunhofer.de (H.S.); felix.kuke@iap.fraunhofer.de (F.K.); oliver.dorn@b-tu.de (O.N.D.); janina.zoglauer@tu-braunschweig.de (J.Z.); noahkremp@outlook.de (N.K.); 2 Chair of Polymer-Based Lightweight Design, Brandenburg University of Technology, 03046 Cottbus, Germany; 3 Research Division Biopolymers, Fraunhofer Institute for Applied Polymer Research IAP, 14476 Potsdam, Germany; tobias.hueckstaedt@iap.fraunhofer.de; 4 Department Microstructure and Residual Stress Analysis, Helmholtz-Zentrum für Materialien und Energie Berlin, 14109 Berlin, Germany; paul.kamm@helmholtz-berlin.de (P.H.K.); garcia-moreno@helmholtz-berlin.de (F.G.-M.); 5 Department Fiber Composite Material Technologies, Technical University of Applied Sciences Wildau, 15745 Wildau, Germany; 6 Chair of Micro and Precision Devices, Technische Universität Berlin, 10587 Berlin, Germany; dirk.oberschmidt@tu-berlin.de

**Keywords:** metal effect pigment, flowline, weld line, tetrahedral polymer particle, injection molding

## Abstract

This publication presents an improved manufacturing method for tetrahedral metal effect pigment particles that demonstrates reduced flowlines in injection-molded polymer components compared with conventional platelet-shaped pigment particles. The previously published cold forming process for tetrahedral particles, made entirely from aluminum, faced manufacturing challenges, resulting in a high reject rate due to particle adhesion to the micro-structured mold roller. In contrast, this study introduces a new manufacturing method for tetrahedral particles, now consisting of metallized UV-cured thermoset polymer. These particles, dispersed in amorphous matrix thermoplastics, have shown to maintain their shape during the injection molding process. The manufacturing technique for these novel particles is based on UV imprint lithography, omitting the reject rates compared with the previously presented cold rolling process of tetrahedral full aluminum particles. Thus, the novel manufacturing technique for tetrahedral pigment particles shows increased potential for automation through roll-to-roll manufacturing in the future.

## 1. Introduction

Injection-molded thermoplastic parts aiming for esthetic appearance often require an optical metallic effect. However, injection molding of metal effect pigment-filled thermoplastics usually causes appearance defects of the metallic effect known as flowlines. Especially in melt flow confluence areas called weld lines, these flowlines occur. In previous studies [1,2], Demski et al. found the unwanted orientation of conventionally platelet-shaped metallic pigment particles not parallel to the surface of PMMA parts to be the major cause for flowlines in the weld line region, as particle depletion was not observed using computer tomography (CT).

**Figure 1:**
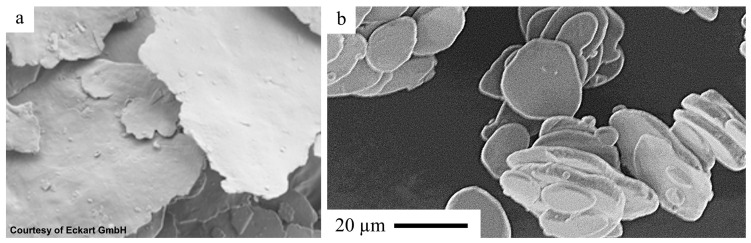
Metal effect pigment particles (SEM image) [1]. (**a**) Cornflake geometry; (**b**) silver dollar geometry (Eckart Reflexal 35).

**Figure 2:**
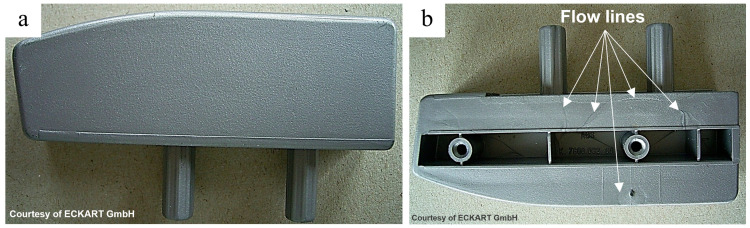
Flowlines on an injection-molded part of metal effect pigment-filled polymer [1]. (**a**) front view; (**b**) back view. Reprinted with permission from Elsevier. Copyright 2025 Elsevier.

The platelet geometry (Figure 1) is still dominant on the market for metal effect pigments. That is why during the design phase of injection-molded parts with emphasis on esthetics, time-consuming simulations have to be conducted to predict flowline formation. Various approaches have been documented, including an approach by Park et al. [3] using the Folgar–Tucker model initially developed for short fiber composites [4], introducing an orientation-related index for surface appearance assessment. Sasayama et al. [5] have developed a simulation using a reverse-time calculation approach, successfully predicting the pigment particle orientation in the surface layer of injection molded parts. Resulting design changes to hide flowlines, e.g., by shifting them to the back of a part (Figure 2), lead to limitations in the final geometry of the polymer parts and costly modifications, which diminish the benefits of injection molding and extrusion in mass production.

Various strategies have been explored to minimize flowlines in injection molding. Santos et al. [6] demonstrated that lowering the mold temperature can lead to flowline reduction in simple mold geometries. However, this approach can also lead to increased visual inconsistencies in other parts of the molded surface. The Shear Controlled Orientation Injection Molding (SCORIM) process, used in conjunction with the Bright Surface Molding (BSM) process [7], can help reduce flowlines by oscillating the polymer melt during cooling. Nevertheless, both SCORIM and BSM necessitate considerable adjustments to the injection mold and machinery, and there are limitations regarding the shape of the molding cavity. Furthermore, alternative pigment shapes have been reported to have an effect on flowline visibility. Lim et al. [8] proposed the use of aluminum platelets with increased thickness; however, the flowline could not be omitted completely, especially for higher filling ratios. Lim et al. [9] proposed the addition of a spherical filler in addition to conventional platelet-shaped pigment particles, which, however, led to higher filling ratios. By modifying the shape of pigment particles from the conventional platelet to novel tetrahedron geometry, Demski et al. [1] achieved a uniform metallic appearance in the weld line region, thus omitting the flowline due to the random orientation of the tetrahedral particles as opposed to the observed pronounced orientation gradients at the weld line. However, manufacturing tetrahedral pigment particles in high quantities by the described roll-to-roll-based cold forming technique from aluminum foil proved to be challenging. The reason was the difficult removal of the particles from the tetrahedral cavities in the roller surface, resulting in a high particle reject rate of about 44%.

The focus of the present study is the development of an improved manufacturing method for tetrahedral metal effect pigment particles for polymeric molding compounds offering a decreased particle reject rate. The process also aims for a narrow particle size distribution, omitting previously necessary sieving steps. Furthermore, compared with conventional atomized and ball-milled platelet particles, as well as full metal tetrahedrons documented in the previous studies [1,2], the pigment presented in the present study consists mainly of a UV-cured thermoset. This means less energy consumption compared with metal atomizing and a lower particle material density compared with full metal particles, reducing the overall weight of the injection-molded component.

## 2. Materials and Methods

Methods for manufacturing tetrahedral metal microparticles have been recorded only for pure aluminum and tin alloys in prior studies [1,2]. The current publication introduces a soft UVimprint lithography method for tetrahedral metal effect pigment particles consisting of metallized transparent thermoset polymer. The manufacturing process is patented [10]. As in the previous studies [1,2], the particles in this study show the same irregular tetrahedron geometry, as their pyramid height is slightly compressed compared with the regular tetrahedron geometry. However, they are referred to as “tetrahedral” in this study for simplicity.

### 2.1. Assessment of THEICTA as a Suitable Material for Particle Manufacturing

THEICTA (Tris(2-hydroxy ethyl) isocyanurate triacrylate) (SR368, ARKEMA, Colombe, France) was chosen for the manufacturing of tetrahedral pigment particles due to its high glass transition temperature of 270 °C according to the manufacturer’s datasheet [11]. A blend of free radical photoinitiators, 2-Hydroxy-2-methyl-1-phenylpropanone, Bis(2,4,6-Trimethylbenzoyl) phenylphosphine oxide, and ethyl (2,4,6trimethylbenzoyl) phenyl phosphinate (Omnirad 2100, IGM Resins, Waalwijk, Netherlands) was used as the photoinitiator for THEICTA. Mixing took place at a weight ratio of 100:1 using a mixing centrifuge SpeedMixer DAC 400.1 VAC-P, Hauschild, Hamm, Germany.

**Figure 3:**
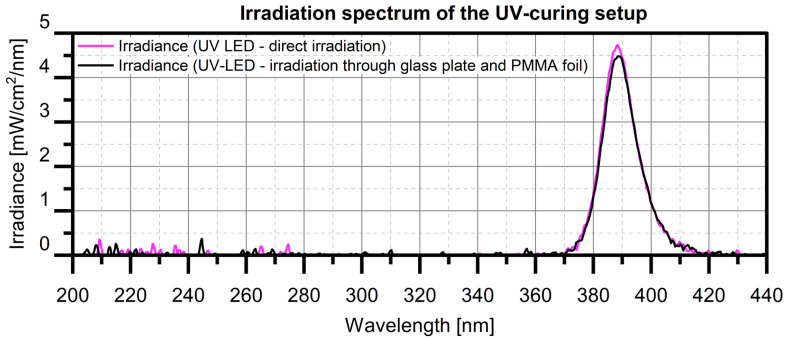
Spectra of the UV LED array.

The characterization of the suitability of THEICTA as a resin for manufacturing tetrahedral filler particles intended for polymethyl methacrylate (PMMA)-based injection molding compounds is described below. UVcuring of test specimens for this characterization is generally conducted using a UV-LED array with a wavelength band between 370 and 420 nm. The spectrum, measured by a handheld UV radiometer UVPAD, Opsytec, Ettlingen, Germany, is shown in Figure 3.

#### 2.1.1. Curing Behavior

In order to achieve the highest possible degree of curing with the above-described UVsource, the ion viscosity of THEICTA during curing was monitored by dielectric analysis (DEA) using a DEA-288-ionic, Netzsch, Selb, Germany. The minimum curing time was identified for specimen thicknesses of 1 mm and 200 µm, where 1 mm corresponded to the thickness of the test specimens for dynamic mechanical analysis (DMA) and mechanical strength measurement (see Section 2.1.3.), while 200 µm corresponded to the approximate height of tetrahedral particles of around 100 µm, including a safety factor of twice the tetrahedron height (see Section 2.2.). DEA was performed while curing THEICTA by UVirradiation through the same cover materials as used later for manufacturing the specimens described in Section 2.1.2, Section 2.1.3 and Section 2.2. The results of the DEA are presented in Section 3.1.1. According to the results, all specimens in this study were cured by UV radiation in the spectrum shown in Figure 3 for 2 min, resulting in a measured dose of 8.79 J cm^−2^ through glass and PMMA foil.

#### 2.1.2. Glass Transition Temperature

The glass transition temperature of the tetrahedral particles should exceed the processing temperature of the intended matrix material during injection molding and dispersion in order to maintain tetrahedral shape. Thus, the glass transition temperature of the UV-cured imprint resin was measured via DMA using an Ares Test Station, Rheometric Scientific, Piscataway, USA in three heating cycles, each with an equal heating rate of 10 K min^−1^. The first cycle indicated the glass transition of UV-cured but untempered THEICTA, and the second cycle was compared with the third cycle as deviations indicated incomplete tempering during the first cycle. The third cycle indicated the glass transition of THEICTA after monitored tempering.

The DMA test specimen was manufactured from THEICTA with dimensions of 10 mm × 30 mm × 1 mm by UVcuring in a rectangular mold covered with a microscope slide using the known setup of lamps described in Section 2.1. The results of the DMA are documented in Section 3.1.2.

#### 2.1.3. Shear Strength

The particles should exceed the solidified matrix material in terms of mechanical strength to maintain their tetrahedral shape during the injection molding process. Especially, the shear subjected to the compound in solid state before melting poses a threat to the filler particles’ shape. Thus, a shear test in line with ASTM D732 [12] was conducted at 23 °C ambient temperature using a tension/compression testing machine 2530-445, Instron, Norwood, USA.

The THEICTA specimens were manufactured in the same manner as the DMA specimens in Section 2.1.2., one batch with and one without a tempering step after UVcuring by heating to 250 °C at 10 K min^−1^ and cooling at the same rate. The THEICTA specimens were molded as circular disks of 1 mm thickness featuring a central hole of 11 mm diameter using a UVtransparent mold with a flexible cylindrical central core in order to decrease internal stress in and around the central hole in the specimen during UVcuring.

PMMA specimens of the same shape were manufactured using a variotherm hot-pressing process at a molding temperature of 190 °C. The results of THEICTA were compared with PMMA, as presented in Section 3.1.3.

#### 2.1.4. Yellowing During Curing

In this study only three surfaces of the tetrahedron were metallized, while the fourth surface was still in contact with the carrier foil for handling. In the application as metallic pigment, light being transmitted through the fourth surface of the particleis transmitted through the tetrahedral particle and reflected at the metallized surface. This necessitates low yellowing of the cured particle material. 

In order to asses potential yellowing of the particle material, the transmission spectra of cured THEICTA mixed with Omnirad 2100 were compared to hot-pressed PMMA foils of 100 µm thickness using an Excellence UV5 spectrometer, Mettler Toledo, Columbus, OH, USA. The spectra are discussed in Section 3.1.4.

### 2.2. Manufacturing of Tetrahedral Thermoset Particles and Subsequent Injection Molding

**Figure 4:**
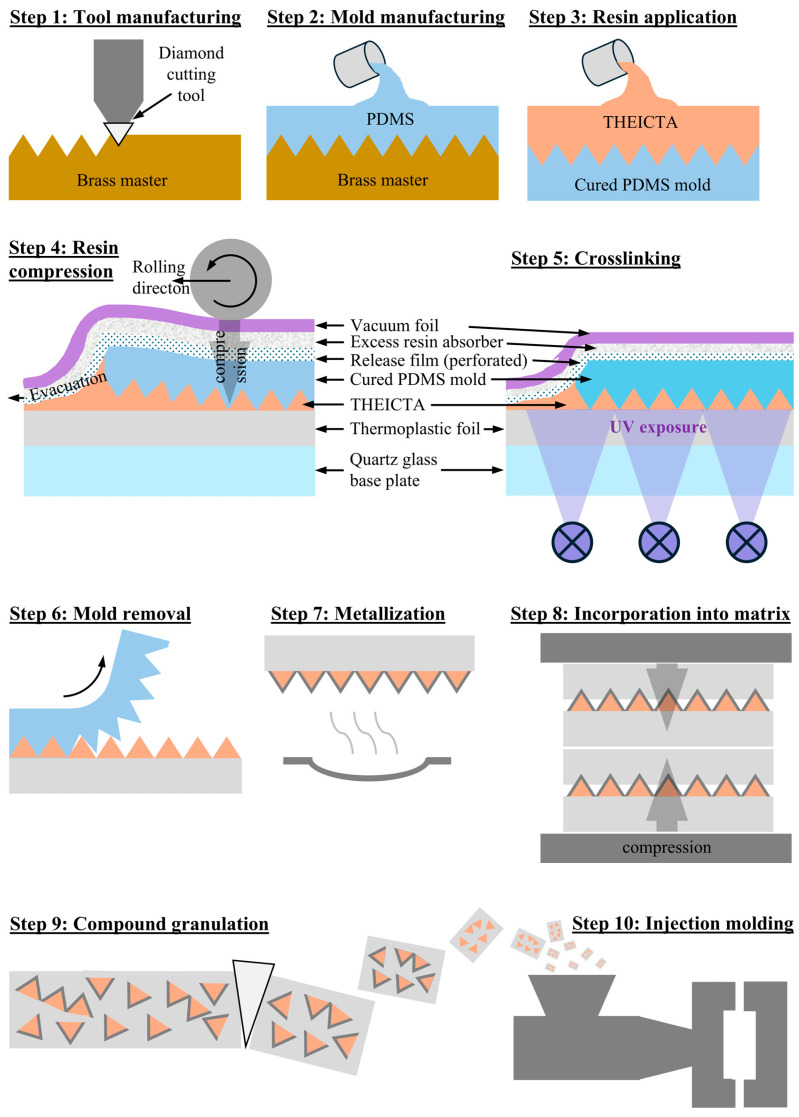
Manufacturing of metallized tetrahedral pigment and injection-molded samples.

Manufacturing the tetrahedral thermoset particles from THEICTA, PVD metallization, dispersion into matrix materials PMMA and polycarbonate (PC), and subsequent injection molding for flowline visibility inspection was conducted applying the following steps as displayed in Figure 4.

**Figure 5:**
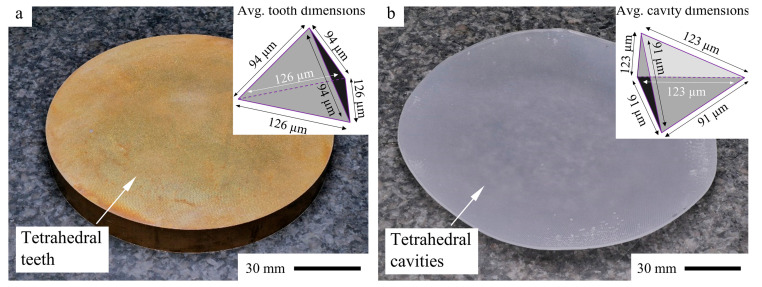
Brass master (**a**) for replication of molds with tetrahedral cavities from PDMS (**b**) [1].

**Step 1—Tool Manufacturing:** A circular brass master structure (Figure 5a) with a microstructure of contiguous tetrahedron-shaped teeth was manufactured during a previous study [1] by shaping on a modified ultra-precision (UP) machining center MMC 1100, LT-Ultra, Herdwangen-Schönach, Germany, using a monocrystalline diamond tool with a 90° corner angle HPHT type 1b, Contour, Valkenswaard, Netherlands. The master structure has a diameter of 100 mm and a thickness of 12 mm.

**Step 2—PDMS Mold Manufacturing:** The mold for manufacturing the tetrahedral THEICTA particles (Figure 5b) was replicated from the brass master structure by gravity casting of addition curing polydimethylsiloxane (PDMS) Elastosil RT 601 and subsequent curing at 100 °C for 20 min.

**Step 3—Resin Application onto the PDMS Mold:** Monomeric THEICTA was heated to 80 °C for decrystallization and mixed under vacuum with photoinitiator Omnirad 2100, at a weight ratio of 100:1 using a centrifuge mixer SpeedMixer DAC 400.1 VAC-P. The monomer/photoinitiator mixture was poured and manually spread on the PDMS mold manufactured in Step 3 and subsequentially degassed at 80 °C under vacuum.

**Step 4—Resin Compression to Form Tetrahedral Particles:** A vacuum bag setup was prepared as follows in order to apply pressure to mold THEICTA into tetrahedral particles and maintain the pressure during curing. To an 8 mm thick quartz glass plate, vacuum foil was adhered using butyl rubber vacuum sealant tape. Between quartz glass and vacuum foil, a stack was placed, consisting of 300 µm thick thermoplastic foil made either of PMMA POQ64, Röhm, Sontheim, Germany, or PC Durabio, Mitsubishi Chemical, Tokyo, Japan, as well as the PDMS mold manufactured in Step 2 with the previously spread monomer/photoinitiator mixture facing the thermoplastic foil, perforated HDPE release film, and non-woven polyester excess resin absorber. A vacuum was applied to compress the described stack and the excess resin was driven out between thermoplastic foil and PDMS mold by manual rolling on top of the vacuum foil using a hand roller.

**Step 5—Crosslinking of THEICTA:** UV radiation of 395 nm was applied to the compressed stack through the quartz glass plate and the thermoplastic foil. According to the DEA results described in Section 3.1.1., the exposure was stopped after 120 s, resulting in a dose of 8.79 J cm^−2^.

**Step 6—Mold Removal:** Removal of the PDMS mold exhibiting self-release properties was performed manually by rolling the flexible mold onto a cylindrical object.

**Step 7—Particle Surface Metallization:** Metallization with a layer of aluminum was conducted at a nitrogen gas pressure of 10^−5^ mbar by thermal evaporation using an evaporation chamber MB EVAP, MBraun, Germany. A molybdenum boat evaporation source and a rotating substrate holder were used. The average layer thickness on a surface vertically oriented to the evaporation source was measured to be 200 nm using a profilometer Dektak 8, Veeco, Plainview, NY, USA; however, as the particle surfaces were inclined with respect to the evaporation source, the aluminum layer thickness was lower but non-transparent to visible light.

**Step 8—Particle Dispersion into Thermoplastic Matrix Polymers and Granulation:** After metallization, several particle-loaded thermoplastic foils made of either PMMA or PC were alternately stacked with non-loaded foils of the same material to aim for a calculated particle volume fraction of 15%. These stacks were pressed at 15 kN and 75% of their respective intended injection screw metering zone temperature (see Table 1) using a hydraulic press KV 241, Rucks, Germany, until the pressure remained constant for 20 s indicating no further melt flow. Subsequent folding and pressing of the resulting hot compound slab were carried out for six iterations each, aiming for particle separation and homogenization of the compound before the following processing steps. The resulting compound was mechanically granulated to an approximately 4 mm grain size.

In addition to the compounds loaded with tetrahedral particles, one compound made of PMMA POQ64 was prepared in the same way as described above, using conventional platelet silver dollar pigment particles Reflexal 100, Eckart, Hartenstein, Germany. This compound serves as the benchmark for comparison of the flowline visibility after injection molding.

**Step 9—Injection Molding: **In order to examine the particle survivability during injection molding and the effect on the resulting flowlines, an injection molding test was conducted. Due to the small amount of compound resulting from the manual pigment manufacturing process, a micro injection molding machine Microsystem 50, Battenfeld, Kottingbrunn, Austria, was used. This machine was chosen in contrast toother micro injection molding machines, due to its 3-zone screw-based plasticizing unit which allowed applying shear to the compound comparable to common injection molding.

**Figure 6:**
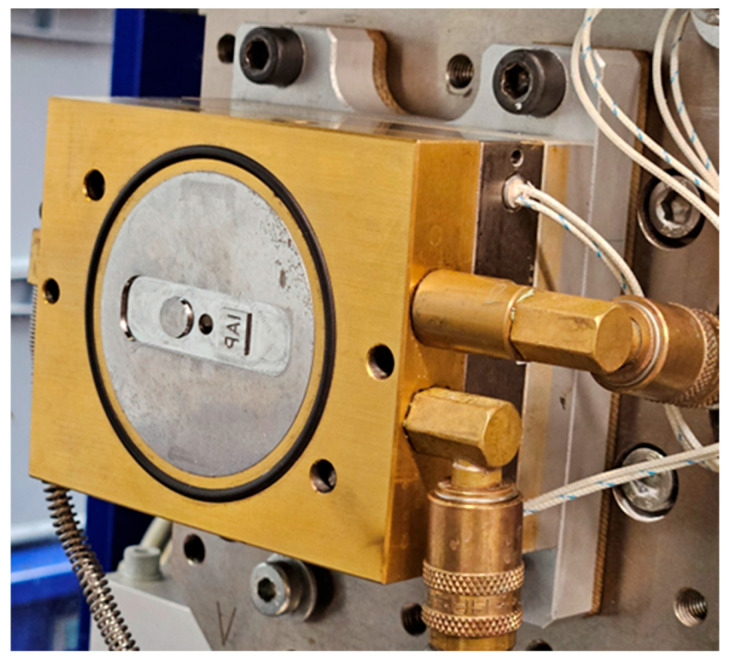
Injection mold (ejector side) with obstacles and central injector pin—injection gate is positioned centrally, opposite the injector pin.

The mold machined from steel for this study featured a cavity of 13 mm × 46 mm × 1 mm with an injection gate in the center and two differently shaped obstacles in opposite directions of the flow (Figure 6). The round obstacle (Ø 8 mm) parted the melt flow fully in two, creating a single flowline, with the square obstacle (7 mm × 8 mm) creating an abrupt decrease in thickness by 0.5 mm in the injection molded part. The granulated compounds were injection molded using the parameters stated in Table 1.

**Table 1:** Thermoplastic matrix materials and injection molding parameters for the pigment compound samples.

Compound No.			Compound 1	Compound 2	Compound 3
**Matrix polymer**			PMMA POQ64	PC Durabio	PMMA POQ64
**Pigment**			Tetrahedral particles	Tetrahedral particles	Reflexal 100 (silver dollar)
**Process** **temperatures**	**Screw**	**Feeding throat [°C]**	19	19	19
		**Compression zone [°C]**	265	245	265
		**Metering zone [°C]**	250	245	250
	**Plungers**	**Metering plunger [°C]**	250	245	250
		**Injection plunger [°C]**	250	257	250
	**Mold**		90	80	90
**Speeds**	**Screw (Ø 14 mm)** **circumferential speed [mm/s]**		20	10	20
	**Injection speed [cm^3^ s^−1^]**		1.96	1.96	1.96

### 2.3. Computer Tomography-Based Observation of the Particles in the FlowLine

The tomography measurements were carried out at the LIMAX-160 instrument at the in situ X-ray lab In-Situ X-Ray Lab operated by Helmholtz-Zentrum Berlin für Materialien und Energie (HZB), Berlin, Germany. The liquid MetalJet X-ray source, Excillum, Sweden was operated at 70 keV and 1000 projections with an exposure time of 500 ms over an angular range of 360° using a CMOS X-ray detector with a 15 µm thick GOS scintillator, Photonic Science, Hastings, UK [13]. The magnification was set to 1.4, resulting in an effective pixel size of 3.2 µm. Due to the high coherence and the low energy regime of the very intense Ga-Kα emission line, it is possible to enhance the contrast by using a single distance phase retrieval algorithm [14]. The corrected projections were reconstructed with X-AID, Mitos, Germany, using the Feldkamp–Davis–Kress algorithm [15].

Image processing was conducted using Amira Software, ThermoFisher Scientific, Waltham, MA, USA. Due to the low absorption contrast of the tetrahedral particles, a VGG16 neural network, trained with 100 epochs, was used for shape recognition of the particles. Subsequently, a combination of watershed, distance transform, and numerical reconstruction operations was conducted for separation of the particles. After labeling, a border kill operation was used to exclude particles cut by the observation border of the CT scan. The resulting 3D-rendered images of the particles are displayed in Figure 12.

## 3. Results and Discussion

### 3.1. Results of the Assessment of THEICTA as a Suitable Material for Particle Manufacturing

As described below, THEICTA has proven suitable in experiments for the manufacturing of tetrahedral filler particles which, in a metallized state, retain their shape in an injection molding process.

#### 3.1.1. Results of the Curing Behavior Assessment

**Figure 7:**
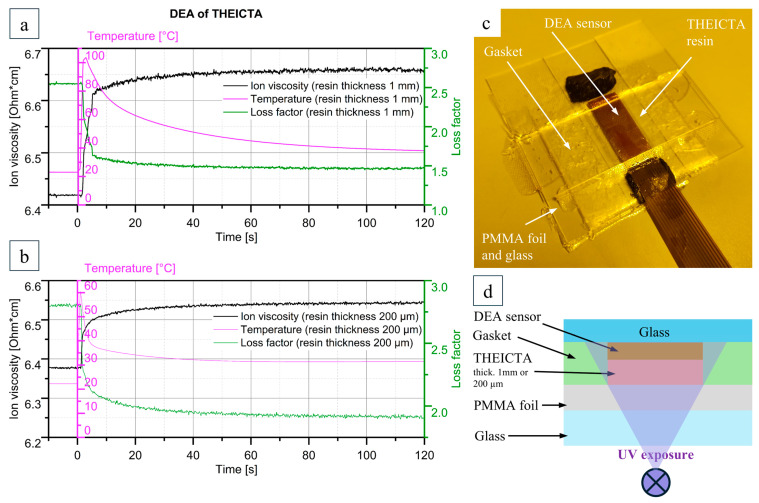
DEA results of THEICTA curing of (**a**) 1 mm and (**b**) 200 µm resin thickness; (**c**,**d**) DEA experiment setup.

Figure 7a,b show the graphs for ion viscosity, loss factor, and resin temperature during curing for 1 mm and 200 µm resin thickness, respectively. The time stamp 0 s marks the beginning of the UV exposure, where a steep increase in ion viscosity and temperature due to the exothermal reaction can be observed. After approximately 100 s, the ion viscosity shows no further increase, indicating the maximum degree of polymerization by UV curing possible with the setup shown in Figure 7c,d. This value sets the limit of UV exposure time for the particle curing process described in Section 2.2., Step 5, as well as for manufacturing specimens for further material characterization, as described in Section 2.1.

Heat treatment after UVcuring resulted in further polymerization, as described in Section 3.1.2.; however, in order to keep the process as economical as possible, keeping the final intent of pigment mass production in mind, the particles in this study were manufactured without any tempering step after UV curing.

#### 3.1.2. Results of the Assessment of the Glass Transition Temperature

**Figure 8:**
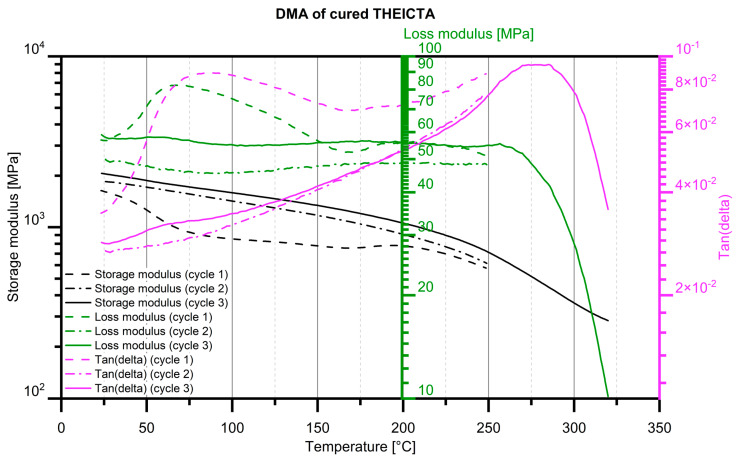
DMA of UV-cured THEICTA.

As shown in Figure 8, the first heating cycle shows that heat treatment after UVcuring resulted in further polymerization. During the first heating cycle, at around 80 °C, the dissipation factor tan (delta) shows a peak, identifying the glass transition temperature of the UV-cured but untempered resin. A local decrease in the storage modulus supports this finding. The second and third heating cycles show similar trajectories, indicating that the resin was fully tempered during the first heating cycle.

During the third heating cycle, a local decrease in the storage modulus could not be identified, indicating the absence of a narrow glass transition region; however, a peak of tan(delta) can be identified at 275 °C, which can be defined as mild glass transition.

These findings indicate that THEICTA could possibly be used in injection molding processes with mass temperatures of close to 300 °C, which include the injection molding of PMMA and PC as matrix material filled with pigment particles made of THEICTA.

#### 3.1.3. Results of the Shear Strength Assessment

The shear strength measured in the experiment described in Section 2.1.3. resulted in the values documented in Table 2. UV-cured but untempered THEICTA shows slightly higher arithmetic mean values of shear strength compared with PMMA, the main intended matrix material for the pigment compound. Tempered THEICTA even shows 33% higher arithmetic mean values of shear strength. The standard deviation of the shear strength of THEICTA is high compared with PMMA, which could result from higher residual stresses of UV-cured THEICTA compared with hot-pressed PMMA. However, this is a study on bulk material, whose results—particularly with respect to residual stress—are not necessarily transferable to microparticles. This is why the particles were examined under injection molding conditions (see Section 2.2 Step 9 and Section 3.2).

The generally higher shear strength values of THEICTA, especially after tempering, give a hint that THEICTA could be a suitable resin for manufacturing pigment particles that retain their shape during injection molding in a PMMA matrix, especially when high shear is subjected to the still unmolten compound by the injection molding screw. However, considering the shear test was conducted on bulk material, it can only give an estimation on the behavior of THEICTA particles in a PMMA matrix subjected to shear in the injection molding process. To fully clarify the suitability of THEICTA, an actual injection molding test, described in Section 2.2. Step 9, was conducted.

#### 3.1.4. Results of the Assessment of Yellowing During Curing

**Figure 9:**
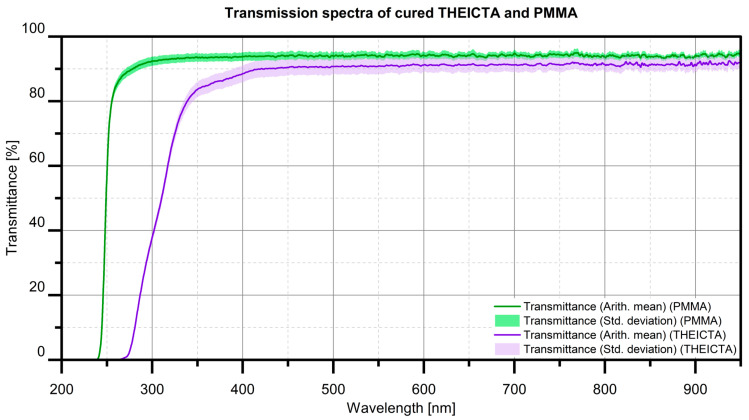
Transmission spectra of cured THEICTA and PMMA.

As displayed in Figure 9, the transmission of visible light through 100 µm thick material is on average 93% for PMMA and 91% for cured THEICTA, with Omnirad 2100 as the initiator at a 100:1 ratio. As the pigment particles’ sizes are in the range of the examined foil thickness, THEICTA is a suitable material for micrometer-sized transparent pigment particles, as light can be reflected on the metallized particle surface after transmitting through the particle. Considerable yellowing was not observed for thin foils of 100 µm thickness.

#### 3.1.5. Manufacturing Results

**Figure 10:**
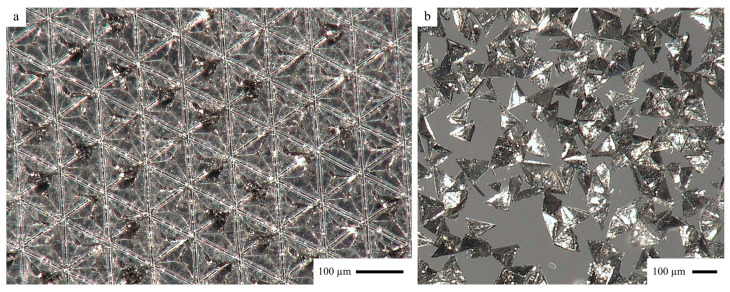
Optical microscope image of tetrahedral THEICTA particles (**a**) on substrate before melting and granulating and (**b**) removed from substrate for examination.

Pigment particle manufacturing according to the process shown in Figure 4 proved to result in particles with very high replication fidelity and sharp edges (see Figure 10). Compared with the manufacturing process for full aluminum tetrahedrons with a reject rate of 44% described in previous studies [1,2], the reject rate for the manufacturing process described in the present study is close to 0%.

All four PDMS molds used in this study lasted for 15 imprint cycles. From the sixteenth cycle on, the diffusion of SR368 into the PDMS surface resulted in slight adhesion of the particles to the mold surface, which is why the PDMS mold was replaced. In future studies, different mold materials will be tested to replace PDMS in order to decrease monomer diffusion into the particle mold by using, e.g., fluorinated elastomers or PTFE.

### 3.2. Results of the FlowLine Visibility inInjection-Molded Parts

**Figure 11:**
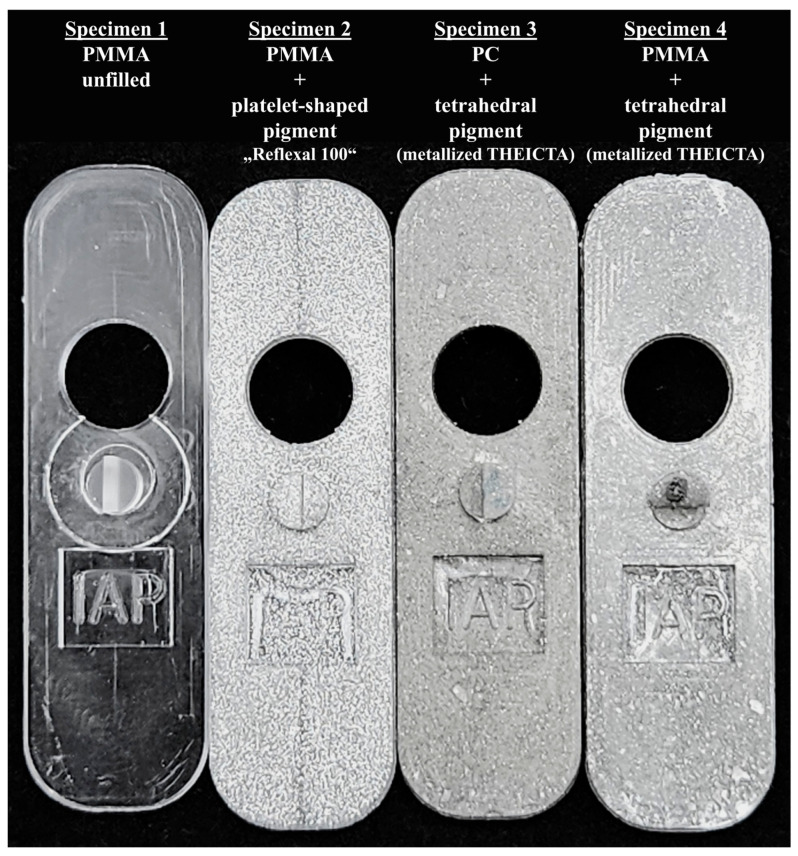
Injection-molded test specimens. The flowline prominent in the platelet-filled specimen is greatly reduced in the tetrahedron-filled specimen.

The injection-molded test specimens manufactured by the process described in Section 2.2. are displayed in Figure 11. Regardless of the displayed matrix material, the samples filled with tetrahedral pigment particles showed significantly reduced flowlines compared with the platelet-shaped pigment. No differences in weld line visibility were observed across all 15 injection-molded samples. The reason for the slight difference in color between the PC and PMMA parts filled with tetrahedral pigment was likely to be an aluminum oxide layer due to the reaction of oxygen on the polymer surface and aluminum during PVD. This was optimized in the case of the PMMA samples by storing in a nitrogen atmosphere for 24 h before PVD.

### 3.3. Results of the Computer Tomography-Based Observation of the Particles in the FlowLine

**Figure 12:**
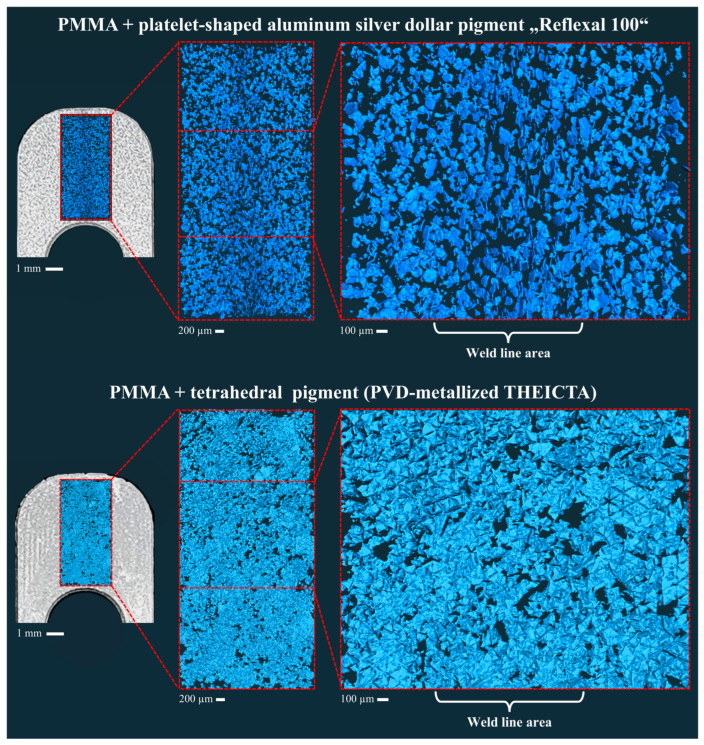
Three-dimensional-rendered images of the particles obtained by X-ray tomography.

In Figure 12, the same orientation behavior of platelet and tetrahedral particles can be observed in the weld line as described for full aluminum particles in the previous studies [1,2]. While the platelet-shaped particles cause flowlines in specimen 2, the tetrahedral particles show homogenous orientation, which leads to the more homogenous optical metallic effect displayed in Figure 11, specimen 3 and 4. This proves the corresponding behavior of the metallized THEICTA tetrahedrons and the full aluminum tetrahedrons described in [1,2]. However, the mesh generated from the CT data shows that not all particles were separated from one another by the shear subjected to the compound during the injection molding process. This corresponds with the irregular reflective speckles of the tetrahedral pigment in Figure 11, at the lower edge of specimen 3 and 4. This is why future studies will focus on the optimization of particle separation.

## 4. Conclusions and Outlook

This study demonstrates the successful development and application of a novel soft UVimprint lithography process for manufacturing tetrahedral metal effect pigment particles made of UV-cured and subsequently vacuum-metallized THEICTA. Compared with conventional platelet-shaped pigments, the tetrahedral particles show significantly reduced flowline visibility in injection-molded PMMA and PC matrix materials, resulting in a more homogeneous metallic appearance of injection-molded parts. This corresponds with findings from previous studies with full aluminum tetrahedral particles; however, the pigment presented in this study has a lower particle mass, while its manufacturing process shows a negligible reject rate.

The pigment exhibits suitable mechanical, thermal, and optical properties with PMMA and PC matrix materials in injection molding processes, as experimentally proven for bulk material and in the actual process.

Some particle agglomeration remains, leading to minor optical inhomogeneities in the form of reflective speckles. Thus, future work will focus on optimizing particle separation and on the automation of the particle manufacturing process using a roll-to-roll approach with a roller-shaped master structure and continuous curing of particles.

## Figures and Tables

**Table 2:** Shear strength values of PMMA and THEICTA bulk specimens.

Specimen	No. 1	No. 2	No. 3	No. 4	No. 5	Arith. Mean	Std. dev.
**Shear strength PMMA [MPa]**	67.11	66.38	67.09	68.02	62.10	66.14	2.09
**Shear strength THEICTA untempered [MPa]**	48.69	42.35	69.01	98.09	85.98	68.83	21.25
**Shear strength THEICTA tempered [MPa]**	73.73	100.84	82.98	84.48	154.27	99.25	28.85

## Data Availability

The original contributions presented in this study are included in the article. Further inquiries can be directed to the corresponding authors.

## Abbreviations

The following abbreviations are used in this manuscript:

BSM	Bright Surface Molding (an injection molding process)
CT	Computer Tomography
DEA	Dielectric Analysis
DMA	Dynamic Mechanical Analysis
PC	Polycarbonate
PDMS	Polydimethylsiloxane
PMMA	Polymethylmethacrylate
SCORIM	Shear Controlled Orientation Injection Molding (an injection molding process)
SEM	Scanning Electron Microscopy
THEICTA	Tris(2-hydroxy ethyl) isocyanurate triacrylate
